# Supplementary material for: Mechanisms governing the pioneering and redistribution capabilities of the non-classical pioneer PU.1
Source: Nat Commun. 2020 Jan 21;11:402. doi: 10.1038/s41467-019-13960-2 (PMC6972792; doi:10.1038/s41467-019-13960-2)
Supplement: Supplementary file 7 — Source data [file 41467_2019_13960_MOESM7_ESM.zip › Source_Data/FigureS2/FigureS2B_MotifScanOutput/KmeansCluster1/homerResults/motif24.similar.html]

motif24

## Information for motif24

A
C
G
T
A
G
C
T
T
A
G
C
G
C
T
A
G
T
C
A
G
T
A
C
G
C
T
A
A
C
G
T
C
G
T
A
T
C
A
G
C
G
A
T
T
C
G
A
A
G
T
C
  
Reverse Opposite:  

T
C
A
G
A
C
G
T
G
C
T
A
A
G
T
C
G
C
A
T
T
G
C
A
C
A
G
T
C
A
T
G
A
C
G
T
C
G
A
T
A
C
T
G
C
T
G
A
T
C
G
A
  

|  |  |
| --- | --- |
| p-value: | 1e-33 |
| log p-value: | -7.627e+01 |
| Information Content per bp: | 1.827 |
| Number of Target Sequences with motif | 68.0 |
| Percentage of Target Sequences with motif | 1.34% |
| Number of Background Sequences with motif | 50.5 |
| Percentage of Background Sequences with motif | 0.13% |
| Average Position of motif in Targets | 100.5 +/- 69.2bp |
| Average Position of motif in Background | 106.4 +/- 63.2bp |
| Strand Bias (log2 ratio + to - strand density) | 0.0 |
| Multiplicity (# of sites on avg that occur together) | 1.00 |
| Motif File: | file (matrix) reverse opposite |

### Similar de novo motifs found

|  |  |  |  |  |  |  |  |
| --- | --- | --- | --- | --- | --- | --- | --- |
| Rank | Match Score | Redundant Motif | P-value | log P-value | % of Targets | % of Background | Motif file |
| 1 | 0.960 | G A C T G A T C G C T A G T C A A G T C C T G A A G C T C G T A T C A G C A G T T C G A A G T C | 1e-32 | -73.909645 | 1.32% | 0.13% | motif file (matrix) |
| 2 | 0.896 | T C A G G A C T C G T A A G T C G C A T T C G A C G A T T C A G A C G T C A G T C T A G | 1e-31 | -71.990002 | 1.55% | 0.21% | motif file (matrix) |
| 3 | 0.876 | G T C A C G T A A G T C C T G A A G C T C T G A C T A G C G A T C T G A A G T C | 1e-25 | -58.995466 | 1.69% | 0.34% | motif file (matrix) |
| 4 | 0.829 | C G T A A G T C C T G A A C G T C G T A A C T G A C G T C G T A A G T C | 1e-16 | -37.240425 | 1.24% | 0.30% | motif file (matrix) |
